# Anti-Neuroinflammatory Effect of Jaeumganghwa-Tang in an Animal Model of Amyotrophic Lateral Sclerosis

**DOI:** 10.1155/2019/1893526

**Published:** 2019-02-12

**Authors:** Sun Hwa Lee, Eun Jin Yang

**Affiliations:** Department of Clinical Research, Korea Institute of Oriental Medicine, 1672 Yuseong-daero, Yuseong-gu, Daejeon 305-811, Republic of Korea

## Abstract

Neuroinflammation is considered a critical factor in the pathologic mechanisms of amyotrophic lateral sclerosis (ALS). This study examined the levels of neuroinflammatory proteins in the spinal cord of JGT-treated* hSOD1*^*G93A*^ transgenic mice to determine the effect of Jaeumganghwa-Tang (JGT) on neuroinflammation. Twelve 8-week-old male experimental mice were randomly allocated to three groups: a non-transgenic group, a hSOD1^G93A^ transgenic group, and a hSOD1^G93A^ transgenic group that received JGT 1 mg/g orally once daily for 6 weeks. After 6 weeks, the spinal cord tissues were analyzed for inflammatory proteins (Iba-1, toll-like receptor 4, and tumor necrosis factor-*α*) and oxidative stress-related proteins (transferrin, ferritin, HO1, and NQO1) by Western blot analysis. Administration of JGT significantly delayed motor function impairment and reduced oxidative stress in* hSOD1*^*G93A*^ transgenic mice. JGT effectively ameliorated neuroinflammation mechanisms by downregulating TLR4-related signaling proteins and improving iron homeostasis in the spinal cord of* hSOD1*^*G93A*^ mice. JGT could help to decrease neuroinflammation and protect neuronal cells by strengthening the immune response in the central nervous system. This is the first study to demonstrate the role of JGT in neuroinflammation in an animal model of ALS.

## 1. Introduction

Amyotrophic lateral sclerosis (ALS) is a rapidly progressive neurodegenerative disorder characterized by loss of upper and lower motor neurons in the brain and spinal cord, leading to muscle atrophy, paralysis, and death, usually within 3–5 years of diagnosis [1]. Most patients have sporadic ALS (sALS), whereas 5%–10% have familial ALS (fALS); in the latter group, 20% of cases are caused by mutations in the gene encoding for Cu/Zn superoxide dismutase 1 (*SOD1*) [2]. This mutation is thought to induce expression and aggregation of a toxic gain-of-function protein. Thus far, the* SOD1*, TAR DNA-binding protein 43 (*TDP-43*), Alain (*Als2*), fused in sarcoma (*FUS*), optineurin (*OPTN*), Ubiquilin2 (*UBQLN2*), and* C9ORF72* genetic mutations [3–8] have been investigated for their causal relationship with ALS.

The human mutant* SOD1* transgenic mouse is extensively used as an animal model of fALS. This mouse ubiquitously expresses the human* SOD1* transgene and has a glycine-to-alanine substitution at codon 93 (*hSOD1*^*G93A*^) [9]. Transgenic* hSOD1*^*G93A*^ mice display progressive degeneration of motor neurons similar to that observed in humans with ALS [10]. Although the cause of this degeneration is unclear in patients with ALS, multiple cellular pathogenetic events, such as excitotoxicity, autoimmunity, oxidative stress, and neuroinflammation in motor neurons, have been demonstrated in transgenic* hSOD1*^*G93A *^mice and may be involved in the development of ALS [11–15]. Neuroinflammation is an immune response characterized by neurodegenerative changes and is associated with immune disorders in the central nervous system (CNS), and it contributes to loss of neurons and disease progression in many neurologic diseases. In general, inflammation in ALS is characterized by accumulation of activated microglia and astrocytes as well as gliosis. Activation of microglial cells in ALS has been extensively characterized and is marked by elevated production of potentially cytotoxic molecules, inflammatory mediators, and proinflammatory cytokines, such as tumor necrosis factor-*α* (TNF-*α*) in the toll-like receptor 4 (TLR4) signaling pathway [15, 16]. These molecules can cause further neuronal cell damage and induce activation of microglial cells, resulting in a positive response to neuroinflammation. In addition, the pivotal role of antioxidative stress mechanisms in the complex defense against reactive oxygen species (ROS) produced by cells during normal cellular metabolism suggests that oxidative stress plays a role in the pathogenesis of ALS. Postmortem analysis of tissue from patients with fALS and sALS has shown extensive oxidative damage to proteins, lipids, and DNA. In addition, transgenic mice expressing the mutant human* SOD1* form show clear signs of increased oxidative stress-related proteins and lipid oxidation [17].

Although riluzole is currently approved by the US Food and Drug Administration for use in patients with ALS and prolongs survival by about 3 months, it is very expensive and its side effects are too severe for the short extension of lifespan that it provides. Recently, edaravone was approved by the US Food and Drug Administration as an antioxidant for treatment of ALS patients based on the results of the Edaravone (MCI-186) ALS 19 Study Group [18]. However, this drug has been tested in ALS patients diagnosed at an early stage and there have been adverse effects reported such as renal impairment. In addition, there is no survival data available yet for patients treated with edaravone. As such, there are still no effective treatments for ALS [19] and new therapies that can slow disease progression with less severe side effects are urgently required.

The National Center for Complementary and Integrative Health defines complementary and alternative medicine (CAM) as a diverse set of medical and health care systems, practices, and products that are not considered a part of general medicine [20]. In recent years, research on CAM, including herbal medicines, acupuncture, yoga, meditation, and diet therapy, has been increasing in the quest for potential treatments for ALS. Pagnini* et al*. reported that symptoms of anxiety and depression and negative emotions improved in patients with ALS after an 8-week ALS-specific meditation program [21]. Zhao* et al*. reported that ketogenic diet-fed mice showed increased body weight, slower impairment of motor performance, and a higher number of motor neurons compared with controls [22]. Furthermore, other studies reported that bee venom and* Scolopendra subspinipes mutilans* attenuated neuroinflammation in the spinal cords of symptomatic* hSOD1*^*G93A*^ transgenic mice [23, 24]. All the above-mentioned studies suggest that CAM therapy could improve motor function and increase the lifespan of patients with ALS.

Jaeumganghwa-Tang (JGT,* Zi-yin-jiang-huo-tang* in Chinese,* Jiin-koka-to* in Japanese), a CAM therapy, is a traditional oriental herbal medicine that consists of 12 medicinal herbs [25]. In the Dongui Bogam, a Korean medical text, JGT is reported to have pharmacologic effects that ameliorate night sweats, coughing, fever in the afternoon, and hemoptysis [26]. Clinically, JGT is useful for the treatment of acute chronic bronchitis, upper respiratory tract infections, pulmonary tuberculosis, and bronchial asthma [27]. Kim* et al*. reported that JGT may have anticancer effects because of its ability to inhibit secretion of inflammatory cytokines such as TNF-*α* and interleukin-6 (IL-6) in human mast cells by blocking activation of nuclear factor (NF)-*Κ*b [26]. Further, Zheng* et al*. reported that JGT reduces the incidence and severity of hot flushes caused by tamoxifen in patients with breast cancer [28]. However, no studies have thus far reported on the antineuroinflammatory effects of JGT in neurodegenerative disease. The aim of this study was to determine if JGT could attenuate neuroinflammation in the spinal cord in an animal model of ALS.

## 2. Materials and Methods

### 2.1. Animals

All mice used in this experiment were treated in accordance with the guidelines published by the United States National Institutes of Health (Bethesda, MD, USA). The experimental procedures were approved by the Institutional Animal Care and Use Committees of the Korea Institute of Oriental Medicine (reference number #15-036).

Eight-week-old male hemizygous transgenic B6SJL mice carrying a glycine-to-alanine mutation at codon 93 in the cytosolic Cu/Zn superoxide dismutase gene (*hSOD1*^*G93A*^) were sourced from the Jackson Laboratory (Bar Harbor, ME, USA). The transgenic status of the mice was confirmed by polymerase chain reaction (PCR) as described previously [29]. All mice were maintained under the same standard housing conditions, with free access to water and standard rodent chow obtained from Orient Bio (Gyeonggi-do, Korea).

### 2.2. JGT Treatment

JGT was purchased from Han Kook Shin Yak Pharmaceutical Co., Ltd. (Chungnam, Korea), and diluted with autoclaved distilled water. The mice were randomly allocated to three groups: a non-transgenic group, a hSOD1^G93A^ transgenic group, and a hSOD1^G93A^ transgenic group that received JGT 1 mg/g orally once daily for 6 weeks.

### 2.3. Western Blot Analysis

After 6 weeks, the mice were euthanized with pentobarbital. The spinal cords were dissected and homogenized in RIPA buffer (50 mM Tris-Cl, pH 7.4, 1% NP-40, 0.1% sodium dodecyl sulfate [SDS], and 150 mM NaCl) containing a protease and phosphatase inhibitor cocktail (Thermo Fisher Scientific, Rockford, IL, USA). The homogenized tissue was centrifuged at 19.083* g* for 20 min at 4°C. Total protein was quantified using the bicinchoninic acid assay kit (Pierce Biotechnology Inc., Rockford, IL, USA). Samples denatured in SDS sampling buffer were separated by SDS-polyacrylamide gel electrophoresis and transferred to a polyvinylidene difluoride membrane (Bio-Rad, Hercules, CA, USA) for Western blotting. For detection of target proteins, the membranes were blocked with 5% skim milk (Sigma-Aldrich, St. Louis, MO, USA) in Tris-buffered saline and incubated with various primary antibodies, including anti-tubulin, anti-HO1, anti-ferritin, and anti-TNF-*α* (Abcam, Cambridge, UK; 1:1000), anti-Iba-1 (Wako, Osaka, Japan; 1:1000), and anti-TLR4, anti-transferrin, anti-BAX, and anti-NQO1 (Santa Cruz Biotechnology Inc., Santa Cruz, CA, USA; 1:1000). Subsequently, the blots were probed with horseradish peroxidase-conjugated antibodies (Santa Cruz Biotechnology) and visualized using Femto (Thermo Fisher Scientific). A ChemiDoc image analyzer (Bio-Rad) was used to detect immunoblotted bands.

### 2.4. Footprint Test

The day before the mice sacrifice, we performed a footprint test to analyze motor function. The footprint test determines the extent of muscle loosening by measuring stride length of the mice [30, 31]. This experiment was carried out as previously described [32]. The stride length indicates the average length of the stride measured from the center of each footprint.

### 2.5. Statistical Analysis

All the data were analyzed using GraphPad Prism version 5.0 software (GraphPad Software Inc., San Diego, CA, USA) and are presented as the mean ± standard error of the mean. The Western blot results were analyzed using one-way analysis of variance followed by Newman-Keuls tests. A p-value < 0.05 was considered statistically significant.

## 3. Results

### 3.1. JGT Treatment Improves Motor Functions in *hSOD*1^*G*93*A*^ Mice

**Figure 1:**
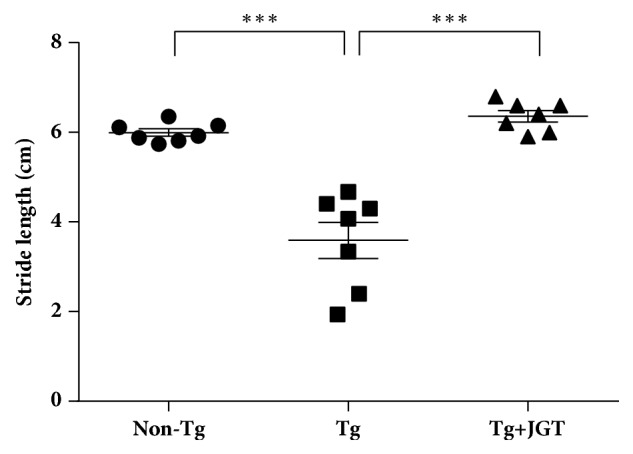
Effect of JGT on motor functions of hSOD1^G93A^ mice. Footprint tests were performed to determine the effect of JGT on the motor function of mice. Comparison of footprint test between groups. Non-transgenic mice (Non-Tg), hSOD1^G93A^ mice (Tg), and JGT-treated hSOD1^G93A^ mice (Tg + JGT). Quantification of stride length from each group. Data are expressed as the mean±SEM (n=7, *∗∗∗*p<0.001).

To investigate the effect of JGT treatment on motor function of hSOD1^G93A^ transgenic mice, we conducted the footprint test as a behavioral test. As shown in Figure 1, the stride lengths of hSOD1^G93A^ transgenic mice (Tg) were 1.6-fold lower (4.12 ± 0.43 cm) than that of non-transgenic mice (Non-Tg) (6.41 ± 0.17 cm) (p < 0.001). However, JGT treatment significantly improved the stride length (6.36 ± 0.13 cm) compared to that of Tg mice (4.12 ± 0.43 cm) (p < 0.001). This finding suggests that JGT treatment could delay motor function impairment in hSOD1^G93A^ transgenic mice.

### 3.2. JGT Reduces Expression of Neuroinflammatory Proteins in the Spinal Cord of the *hSOD*1^*G*93*A*^ Mouse

**Figure 2:**
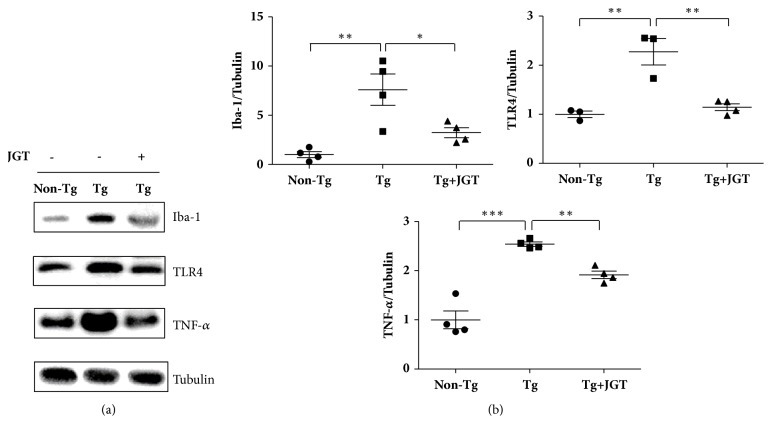
Effect of JGT on neuroinflammation in the spinal cord of hSOD1^G93A^ mice. Spinal cord lysates from non-transgenic mice (Non-Tg), hSOD1^G93A^ mice (Tg), and JGT-treated hSOD1^G93A^ mice (Tg + JGT) were immunoblotted with anti-Iba-1, anti-TLR4, and anti-TNF-*α* (a). Iba-1, TLR4, and TNF-*α* expression levels are higher in the spinal cord of transgenic mice than in the controls. JGT administration reduces the expression of Iba-1, TLR4, and TNF-*α* in the spinal cord compared with transgenic mice. Tubulin is used as a loading control. Quantification of immunoblots of Iba-1, TLR4, and TNF-*α* (b). Data are presented as mean ± standard error of mean (*∗* p < 0.05; *∗∗* p < 0.01; *∗∗∗* p < 0.001). JGT, Jaeumganghwa-tang.

There were significant increases in Iba-1, TLR4, and TNF-*α* expression levels of 7.6-fold, 2.3-fold, and 2.5-fold, respectively, in the spinal cords of* hSOD1*^*G93A*^ transgenic mice when compared with non-transgenic mice; however, treatment with JGT significantly reduced the levels of these inflammatory proteins by 2.4-fold, 2.0-fold, and 1.3-fold, respectively, in the spinal cords of symptomatic* hSOD1*^*G93A*^ mice when compared with those in* hSOD1*^*G93A*^ mice (Figures 2(a) and 2(b)). These findings suggest that JGT exerts its anti-inflammatory effects by decreasing the numbers of microglial cells and expression levels of TLR4-related signaling proteins in the spinal cord of the hSOD1^G93A^ mouse.

### 3.3. JGT Attenuates Oxidative Stress in the Spinal Cord of the *hSOD*1^*G*93*A*^ Mouse

**Figure 3:**
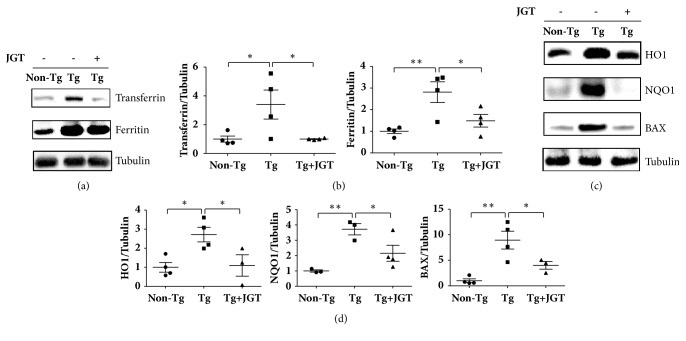
Effect of JGT on oxidative stress-related proteins in the spinal cord of hSOD1^G93A^ mice. Representative images of the expression of transferrin and ferritin in the spinal cord of non-transgenic mice (Non-Tg), transgenic hSOD1^G93A^ mice (Tg), JGT-treated transgenic hSOD1^G93A^ mice (Tg+JGT) (a). Quantification of each immunoblot (b). Tubulin is used as a loading control. Representative immunoblots of HO1, NQO1, and BAX (c). Quantification of each immunoblot (d). Expression of oxidative stress-related proteins is higher in the spinal cord of transgenic mice than in the controls. JGT administration reduces the protein level in the spinal cord compared with transgenic mice. Data are shown as mean ± standard error of mean (*∗* p < 0.05; *∗∗* p < 0.01). JGT, Jaeumganghwa-tang.

Transferrin, ferritin, HO1, and NQO1 protein levels increased by 3.4-fold, 2.8-fold, 2.7-fold, and 3.7-fold, respectively, in the spinal cords of* hSOD1*^*G93A*^ mice when compared with non-transgenic mice; however, administration of JGT significantly decreased expression of transferrin, ferritin, HO1, and NQO1 by 3.4-fold, 1.9-fold, 2.5-fold, and 1.7-fold, respectively, in the spinal cords of* hSOD1*^*G93A*^ mice when compared with transgenic mice (Figures 3(a)–3(d)). As expected, expression of BAX, which plays a role in neuronal cell death, was 8.9-fold higher in the spinal cords of* hSOD1*^*G93A*^ mice when compared with non-transgenic mice; however administration of JGT reduced the expression of BAX by 2.2-fold in the spinal cords of* hSOD1*^*G93A*^ mice (Figures 3(c) and 3(d)). These results suggest that JGT prevents neuronal cell death by oxidative stress in the spinal cord of the* hSOD1*^*G93A*^ mouse.

## 4. Discussion

ALS is a neurodegenerative disease that causes progressive degeneration of motor neurons in the motor cortex, brainstem, and spinal cord. Thus far, the pathologic mechanism of ALS is unclear because of the complex nature of this syndrome, which encompasses both fALS and sALS and involves not only motor neurons but also nonneuronal cells, including astrocytes, microglia, oligodendrocytes, and muscle cells. Given that there is no cure for ALS, research to identify useful therapies is crucial.

CAM therapy, including acupuncture, herbal medicine, and yoga, is popular in view of the significant limitations of conventional therapy, in particular, its side effects. Acupuncture is one of the most popular CAM therapies used by patients with ALS [33, 34]. Pan* et al. *have reported on the CAM therapies used in Shanghai to reduce the side effects of riluzole in patients with ALS [35]. Further, Wasner* et al*. have reported that patients with ALS use a wide range of therapies, including acupuncture, homeopathy, and naturopathy, as well as other more esoteric treatments, to delay progression of the disease [36]. From these reports, we suggest that CAM therapies could help to improve quality of life in patients with ALS.

Neuroinflammation is considered a critical factor in a number of neurodegenerative conditions, including Alzheimer's disease, Parkinson's disease, multiple sclerosis, and ALS [37–39]. Neuroinflammation in the brain is caused by microglia and astrocytes known as glial cells. The microglia help to remove toxic aggregated proteins and cell debris from the CNS. However, activated microglia are toxic to neuronal cells because they release several proinflammatory factors, including TNF-*α* and IL-1*β*, as well as free radicals, including nitric oxide and superoxides [40]. Potenza* et al*. demonstrated that the antineuroinflammatory factor fingolimod phosphate (FTY720), which acts as an immunomodulator, significantly modulated neuroinflammatory and protective genes (*CD11b*,* Foxp3*,* iNOS*,* Il1β*,* Il10*,* Arg1*, and* Bdnf*) by controlling activation of microglia in the motor cortex and spinal cord in animals with ALS [41]. Further, Laura* et al*. reported that MM218, a specific inhibitor of extracellular cyclophilin A, increases the survival time of* hSOD1*^*G93A*^ mice by reducing levels of proinflammatory markers and protecting motor neurons [42].

JGT is a commonly prescribed traditional oriental herb in Eastern countries and is used to strengthen the immune system in patients with acute or chronic bronchitis and those with upper respiratory tract infections [27]. Further, JGT is known to decrease the levels of inflammatory cytokines, including TNF-*α* and IL-6, which suggests a potential anticancer effect. Therefore, in this study, we examined the effect of JGT on neuroinflammation in the spinal cord of* hSOD1*^*G93A*^ mice. Our present findings with regard to neuroinflammation-related proteins suggest that JGT could have antineuroinflammatory effects in neuronal degenerative diseases such as Alzheimer's disease, Parkinson's disease, and multiple sclerosis.

Oxidative stress induced by ROS causes damage to DNA, proteins, and lipids and is involved in the pathogenesis of many neurodegenerative diseases, although whether it is a cause or a consequence is unclear. TLRs are widely expressed in microglia and astrocytes, and upregulation of TLR-related signaling proteins in association with spinal cord damage triggers release of cytokines and chemokines, including ROS [43]. In patients with ALS, systemic changes in the redox and inflammatory states are associated with some clinical parameters [44]. Blasco* et al*. reported that TNF-*α* could trigger oxidative stress via a mechanism involving the NF-*κ*B signaling pathway, which could be dysregulated in ALS [45], suggesting a role of the relationship between oxidative stress and neuroinflammation in the pathogenesis of ALS [46]. BAX is a proapoptotic molecule and Kim et al. showed that JGT fermented with* Lactobacillus acidophilus *increased the expression of BAX causing apoptotic cancer cell death in HT1080, human fibrosarcoma cells [47]. In contrast, our results showed that JGT reduced the expression level of BAX leading to neuroprotective effects in the spinal cord of hSOD1^G93A^ mice. These contradictory results suggest that the composition of fermented JGT and JGT may be different and that the action mechanism of fermented JGT and JGT may also depend on the disease animal model.

The results of the present study with regard to certain oxidative stress-related proteins (transferrin, ferritin, HO1, and NQO1) suggest that JGT could help to reduce neuroinflammation-related events and neuronal cell death in some neurodegenerative diseases.

To our knowledge, this is the first study to demonstrate the role of JGT in neuroinflammation in an animal model of ALS. However, further studies should examine the effect of JGT on muscle tissue in* hSOD1*^*G93A*^ transgenic mice because muscle atrophy and degeneration of motor neurons are important features of ALS as well as the JGT effect on disease onset and progression to support the concept of a therapeutic antineuroinflammatory effect. In addition, the active compound of JGT and its mechanism in the CNS need to be identified. Hence, it is possible that further preclinical assessments of JGT in the context of ALS and other neurodegenerative diseases may reveal that this herb has even greater therapeutic benefits than those suggested in the present study. Such studies have the potential to identify novel therapeutic agents in addition to clarifying the diverse mechanisms of action of closely related nutraceuticals, which will be of great value moving forward.

## 5. Conclusions

In summary, this study shows that JGT ameliorates the levels of proteins involved in neuroinflammation (Iba-1, TLR4, and TNF-*α*) and oxidative stress (transferrin, ferritin, HO1, and NQO1) in the spinal cords of* hSOD1*^*G93A*^ mice. Our findings indicate that JGT could help to reduce neuroinflammation and protect neuronal cells by strengthening the immune response in the CNS.

## Data Availability

The data used to support the findings of this study are included within the article.
